# Effect of thermocycling on flexural strength of dental CAD/CAM ceramics of variable thicknesses and structures: an in vitro study

**DOI:** 10.12688/f1000research.157128.1

**Published:** 2024-10-31

**Authors:** Passent Ellakany, Yousif A. Al-Dulaijan, Nourhan M. Aly, Turki Alshehri, Shahad T. Alameer, Sultan Akhtar, Laila Al Dehailan

**Affiliations:** 1 Division of Prosthodontics, School of Dentistry, The University of Alabama at Birmingham, Birmingham, AL 35209, USA; 2 Department of Substitutive Dental Sciences, Imam Abdulrahman Bin Faisal University, Dammam, Eastern Province, 31441, Saudi Arabia; 3 Pediatric Dentistry and Dental Public Health, Alexandria University Faculty of Dentistry, Alexandria, Alexandria Governorate, 21527, Egypt; 4 Imam Abdulrahman Bin Faisal University College of Dentistry, Dammam, Eastern Province, 31441, Saudi Arabia; 5 Department of Biophysics Research, Institute for Research and Medical Consultations, Imam Abdulrahman Bin Faisal University, Dammam, Eastern Province, 31441, Saudi Arabia; 6 Department of Restorative Dental Sciences, Imam Abdulrahman Bin Faisal University College of Dentistry, Dammam, Eastern Province, 31441, Saudi Arabia

**Keywords:** CAD/CAM, Ceramics, Flexural Strength, Thickness, Composition

## Abstract

**Background:**

This study examined the effect of thermocycling on the flexural strength properties of four CAD/CAM ceramic materials at different thicknesses.

**Methods:**

Four CAD/CAM ceramics of different types: advanced lithium disilicate (ALD), zirconia-reinforced lithium silicate (ZLS) lithium disilicate (LD), and leucite reinforced (LE), and at three varying thicknesses 0.5, 1.0 and 1.5 mm were examined. After subjecting all specimens to 5000 thermomechanical cycles, flexural strength was determined using a universal testing apparatus. Scanning electron microscopy (SEM) was employed for analysis. Two factorial ANOVA models assessed the association of different factors (ceramic type and thickness) with flexural strength and elastic modulus. The 95% confidence intervals (CIs) and adjusted means were computed. A p-value < 0.05 was designated significant.

**Results:**

ZLS exhibited the highest flexural strength at 1.5 mm thickness, while LD showed the highest Young’s modulus of elasticity. The lowest flexural strength was observed in the 0.5 mm thickness group. There were notable variations in flexural strength across all ceramic materials, with the highest adjusted mean strength in the ZLS group, ALD, LD, and LE, respectively. Additionally, significant differences were noted in ceramic thickness, with 1.5 mm thickness showing the highest strength and 0.5 mm thickness the lowest.

**Conclusions:**

Ceramic material thickness significantly impacts flexural strength, with 1.5 mm thickness deemed suitable for posterior restorations. Ceramic materials with zirconia fillers or matrix demonstrated higher flexural strength than other ceramics.

## Introduction

Dental ceramics have evolved to offer restorations with superior esthetic and mechanical characteristics, serving as efficient alternatives to metal-ceramic restorations. ^1^ These dental ceramics can be categorized based on their fabrication method, composition, firing temperature, and microstructure. ^2^ All-ceramic restorations can be fabricated using different methods, including conventional techniques like stacking and sintering, split casting and infusion, and heat- or dry-pressing methods, as well as through computer-aided design and computer-aided manufacturing (CAD/CAM) techniques. ^2, 3^ The homogeneity of ceramic CAD/CAM blocks has notably enhanced the strength of definitive prostheses by reducing crack development and defects compared to conventional ceramic fabrication methods. ^4^ Lithium disilicate glass-ceramic (LD) is one of the most common ceramic materials, with its (SiO _2_-Li _2_O) composition minimizing microcracks and enhancing mechanical properties. ^5, 6^ Previous studies have demonstrated LD’s higher fracture resistance compared to leucite ceramics and improved flexural strength over lithium disilicate-strengthened lithium aluminosilicate glass. ^7, 8^ Leucite glass-ceramic is another option for high-esthetic and translucent all-ceramic restorations, with comparable fracture strength to feldspathic ceramics and resin nano-ceramic. ^9^

Lithium silicate CAD/CAM ceramics reinforced with zirconia (ZLS) integrate tetragonal zirconia fillers to improve ceramic strength, making them capable of withstanding occlusal forces. ^10^ Despite ZLS being challenging to section due to drill blunting, ^11^ its high biaxial flexural strength values have bolstered its utility in fabricating various restorations, including implant-supported molar crowns, occlusal veneers, and endo-crown restorations. ^12^

Advanced lithium disilicate (ALD), a recent addition to the market, comprises lithium disilicate (Li2Si2O5) and virgilite crystals which form a 0.5-μm-long needle-like shape within a zirconia glass matrix. ^13^ Research reporting the mechanical properties of ALD ceramics is scarce. ^14, 15^ One of the published studies showed some positive results regarding the mechanical fatigue behavior of ALD, which is similar to LD but lower than lithium silicate-disilicate and Yttria-stabilized zirconia ^14^ In contrast, another study reported that ALD had lower fracture toughness ^15^ as well as lower flexural strengths when compared to LD. ^16^

The thickness and composition of ceramic restorations have a direct impact on flexural strength and esthetics, where varying restoration thicknesses offer solutions for some clinical challenges. ^17^ For example, thinner restorations can be used for ceramic veneers of high esthetics and translucency, ^18^ while thicker ceramics are more suitable for full-coverage restorations. ^19^ To create a restorative dental material that is highly sustainable, aesthetically pleasing, and safe, all of the material’s qualities must be thoroughly examined and tested. ^20^ Since chewing and biting put occlusal stress on all restorative materials used for tooth restorations, proper flexural strength is considered essential. ^16^ The maximum stress a material can withstand deformation under load is known as flexural strength. ^21^ On the other hand, the minimal and conflicting findings on ALD highlight the need for further studies to comprehensively assess its mechanical properties. ^14– 16^ Additionally, understanding the impact of ceramic thickness and structure can guide the selection of appropriate restoration types for specific dental applications. Thus, this research aimed to evaluate the effect of thermocycling on the flexural strength of four CAD/CAM ceramics of varying thicknesses. According to the null hypothesis, no discernible relationship would be noticed between the flexural strength and the thickness and composition of CAD/CAM ceramics after exposure to thermocycling process.

## Methods

### Grouping of tested specimens

**Figure 1.**
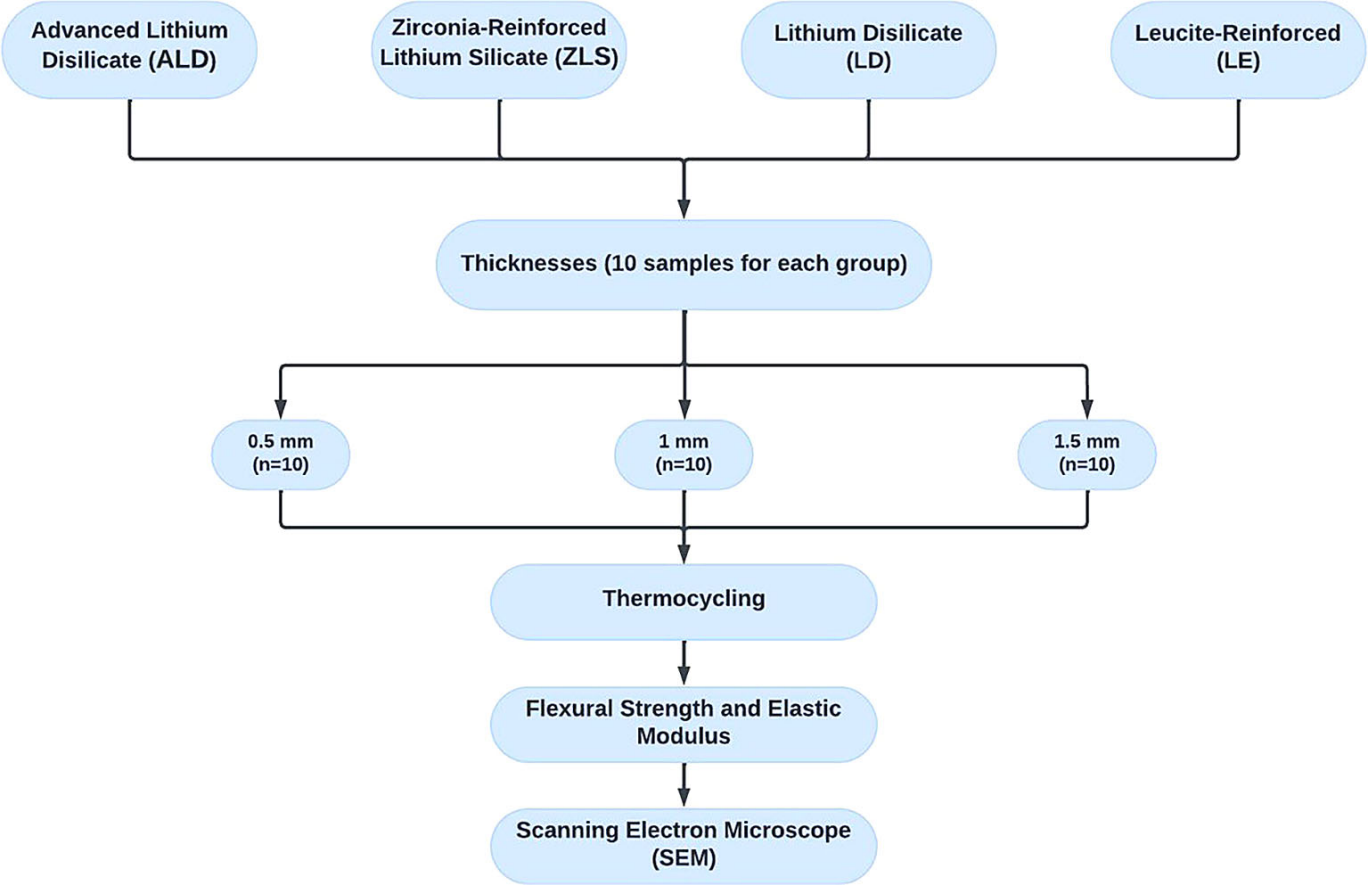
Flow chart representing the study grouping and design.

Four CAD/CAM ceramics of low translucency and A1 shade were examined; advanced lithium disilicate (Cerec Tessera™, Sirona Dentsply, Milford, DE, USA; ALD), zirconia-reinforced lithium silicate (Celtra Duo ^®^, Sirona Dentsply, Milford, DE, USA; ZLS), lithium disilicate (IPS E.max ^®^ CAD, Ivoclar Vivadent, Schaan, Liechtenstein; LD), and leucite reinforced (IPS Empress ^®^ CAD, Ivoclar Vivadent, Schaan, Liechtenstein; LE) as shown in Figure 1. Each ceramic type included 30 specimens, further categorized into three thicknesses of 0.5-, 1-, and 1.5mm. (n=10 specimens per thickness subgroup). As a result, 120 specimens made up the entire sample size that was evaluated in this study. In order to detect an effect size of 0.42, the total sample was computed using G*Power (Version 3.1.9.4), assuming a 5% alpha error and 80% research power. The least number of specimens required for each group was determined to be 9. However, 10 specimens were included to account for possible problems with the laboratory process. ^22^ As a consequence, the number of subgroups multiplied by the number of members in each subgroup yielded 12 × 10 = 120 specimens as the total sample size. ^23^

### Preparation of study specimens

The specimens were sectioned utilizing a precision cutting device (Isomet 5000 machine; Buehler, Lake Bluff, IL, USA) under an abundance of water to produce the following thicknesses of 0.5, 1, and 1.5 mm × 4 mm × 12 mm. Following the specimens’ cutting, a 60-second polishing period was conducted utilizing silicon carbide discs (500-grit coarse) at 200 rpm in the presence of a water-cooling system. The polishing was executed employing a polishing device (MetaServ 250 Grinder-Polisher with Vector Power Head; Buehler, IL, USA). Post-polishing, the specimens were placed in a furnace of ceramic to crystallize. (Programat EP5010; Ivoclar Vivadent, Schann, Liechtenstein). Subsequently, an additional polishing step was performed utilizing the previously described polishing equipment and 400- and 600 grits of carbide discs in a moist atmosphere for 60 seconds at 200 rpm. To ensure dimensional consistency, a digital caliper (Mitutoyo Corp, Kawasaki, Japan) was employed to verify that all specimens maintained a thickness within 0.05 mm. ^23, 24^

### Application of the thermocycling process

The specimens went through a simulated aging protocol, undergoing 5000 thermomechanical cycles in a thermomechanical aging apparatus (Thermocycler THE-1100 machine; SD Mechatronik Feldkirchen, Westerham, Germany). Water baths ranging in temperature from 5°C to 55°C were used for the alternating cycles, with a 10-second interval between each bath and a 30-second immersion period. This simulation replicated the aging impact equivalent to six months of natural aging. ^25– 28^

### Application of flexural strength test

The flexural strength assessment was done using the universal testing machine (Instron 8871 Universal Testing Machine; Instron, Shakopee, MN, USA). A rounded-end steel indenter that was specially made with a 2.5-mm radius was employed. The crosshead speed was set at 1 mm/min, and an axial load of 30 N was directed vertically at the center of the ceramic specimens until fracture happened. ^29^ The maximum load at the point of fracture was noted in Newtons for each specimen. ^25^ Then, megapascals (MPa) were used to calculate the flexural strength based on recommendations outlined in the International Organization for Standardization’s (ISO) 6872 Dentistry—Ceramic Materials. ^30^

Equations listed below were employed to compute the flexural strengths and elastic modulus of specimens.

Flexuralstrength=3FL/2bh2

Where (b) is the tested specimen’s width, (h) is its thickness, (L) is the distance between the two supporting arms, and (F) is the force applied till fracture in Newton. Elastic modulus = FL3/4bh3d where (d) is the deflection measured at (F), and (F) is the load imposed on the linear section of the stress-strain curve (N). ^27^

### Scanning Electron Microscopy (SEM)

For surface qualitative evaluation via scanning electron microscopy (SEM), a randomly selected specimen from each subgroup was chosen to examine the topography of each sample following fracture. The SEM analysis was done using a scanning electron microscope (Inspect S50 model; FEI Company, Moravia, Czech Republic) operating at an increasing voltage of 20 KV and magnifications of ×10000. To reduce the impact of charging and enhance the clarity of the image, the specimens underwent a gold-coating process before examination. ^24^

### Statistical analysis

Plots (Q-Q plots and histograms), normality tests, and descriptive statistics were used to test for normality. All data showed normal distribution, so parametric analysis was adopted. Two factorial ANOVA models were performed to assess the association of different elements (type and thickness of tested ceramics) with flexural strength and elastic modulus. Calculations were done for adjusted means and 95% confidence intervals (CIs). P-value <0.05 was used as the significance threshold. Data analysis was done with Windows-based IBM SPSS (Version 26.0).

## Result

Table 1 presents the flexural strengths and elastic moduli of the four studied ceramics at different thicknesses. At a thickness of 1.5 mm, ZLS exhibited the greatest flexural strength (mean (SD) = 309.08 (33.49)), while LE showed the most minor flexural strength (mean (SD)= 268.11 (7.48)).

**Table 1.** Comparison of flexural strengths and elastic moduli of the four studied ceramics at different thicknesses.

		ALD	ZLS	LD	LE	P value 1
Flexural strength (MPa)	0.5 mm	198.50 (74.59) **A**	223.38 (46.54) **A**	210.65 (27.16) **A**	191.23 (14.41) **A**	0.44
	1 mm	246.95 (48.23) **B**	275.99 (38.41) **B**	267.39 (37.71) **B**	231.45 (40.88) **B**	0.09
	1.5 mm	281.18 (39.51) **ab, B**	309.08 (33.49) **a, B**	301.96 (28.04) **ab, B**	268.11 (7.48) **b, B**	0.01*
	P value 2	0.01*	<0.001*	<0.001*	<0.001*	
Force at break (N)	0.5 mm	90.51 (6.27) **A**	95.76 (31.19) **A**	91.79 (13.13) **A**	85.38 (8.51) **A**	0.63
	1 mm	230.00 (36.49) **ab,** B	258.96 (52.27) **a, B**	251.41 (47.00) **a, B**	195.27 (9.73) **b, B**	0.005*
	1.5 mm	291.36 (67.42) **a, C**	416.56 (74.55) **b, C**	304.40 (85.99) **a, B**	204.04 (15.66) **c, B**	<0.001*
	P value 2	<0.001*	<0.001*	<0.001*	<0.001*	
Young’s modulus of elasticity (GPa)	0.5 mm	22.25 (6.90) **ab, A**	29.51 (7.57) **a, A**	21.95 (7.07) **ab, A**	19.06 (4.72) **b, A**	0.009*
	1 mm	57.28 (16.35) **B**	52.11 (21.02) **B**	48.41 (13.80) **B**	59.65 (45.09) **B**	0.79
	1.5 mm	67.84 (13.81) **ab, B**	82.80 (12.35) **a, C**	67.31 (12.81) **ab, C**	64.07 (15.81) **b, B**	0.02*
	P value 2	<0.001*	<0.001*	<0.001*	0.002*	

a-c: distinct lowercase letters indicate significant variations across groupings. A-C: uppercase letters indicate significant variations in thickness within each group. P value 1: comparison among the various groups. P value 2: comparison of the various thicknesses in every group.

Moreover, at a 1.5 mm thickness, ZLS ceramic required the most significant amount of force to break (mean (SD) = 416.56 (74.55)) in contrast to the same thickness of LE, which required the least amount of force to be broken (mean (SD) = 204.04 (15.66)). Among the 1.5 mm thicknesses, ZLS had the highest Young’s modulus of elasticity (mean (SD)= 82.80 (12.35), while had the lowest (mean (SD)= 82.80 (12.35).

The 0.5mm thickness across all ceramic groups had the lowest flexural strength, elastic modulus, and forces to break. These values were significantly higher at 1.5 mm thickness when compared to 0.5- and 1-mm thicknesses, with ZLS exhibiting the highest values (mean (SD)= 309.08 (33.49), 82.80 (12.35), and 416.56 (74.55)) respectively.

**Figure 2.**
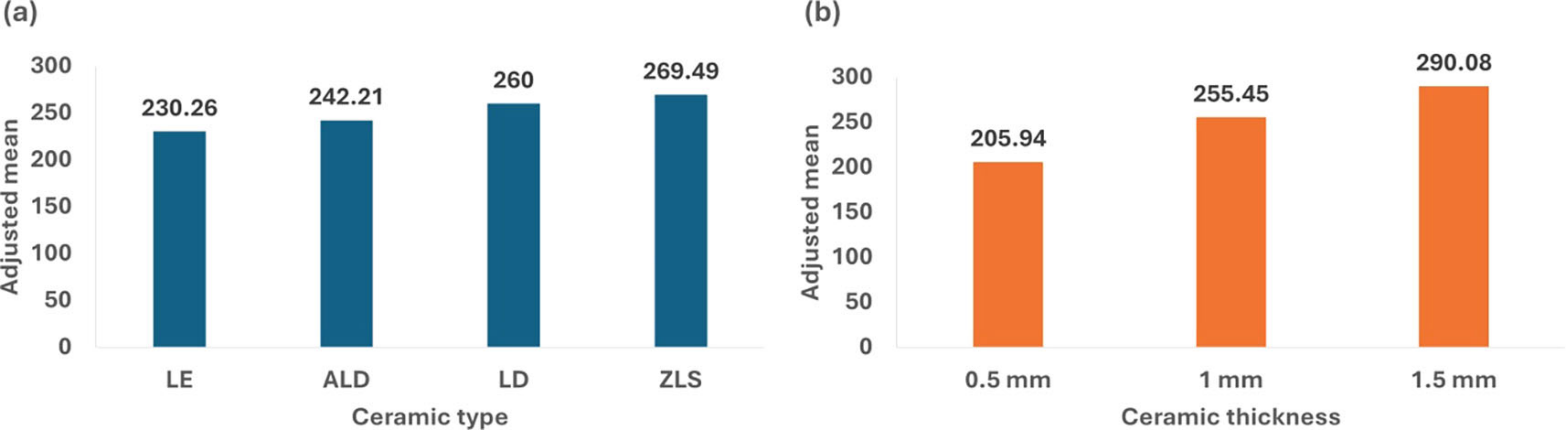
Diagram showing the impact of ceramic type and thickness on flexural strength property.

Table 2 and Figure 2 illustrate the association of flexural strength with ceramic type and thickness. The findings indicated significant variations in flexural strength between the materials, with ZLS exhibiting the highest adjusted mean stress (269.49 MPa), followed by LD (260.00 MPa), ALD (242.21 MPa), and LE (230.26 MPa). Additionally, the materials’ thickness was a major factor, with the 1.5 mm thickness demonstrating the highest strength (290.08 MPa) and the 0.5 mm thickness presenting the least strength (205.94 MPa).

**Table 2.** Factorial ANOVA showing the association of flexural strength with ceramic type and thickness.

		Flexural strength		
		Adjusted mean (SE)	95% CI	P value
Ceramic type	ALD	242.21 (7.03) **a**	228.29, 256.14	0.001*
	ZLS	269.49 (7.03) **b**	255.56, 283.41	
	LD	260.00 (7.03) **ab**	246.08, 273.92	
	LE	230.26 (7.03) **a**	216.34, 244.18	
Thickness	0.5	205.94 (6.09) **a**	193.88, 218.00	<0.001*
	1	255.45 (6.09) **b**	243.39, 267.50	
	1.5	290.08 (6.09) **c**	278.03, 302.14	
Model F (P value)		23.07 (<0.001*)		
Adjusted R ^2^		0.48		

a-c: distinct lowercase letters indicate significant variations across groupings. p value was set significant < 0.05.

**Figure 3.**
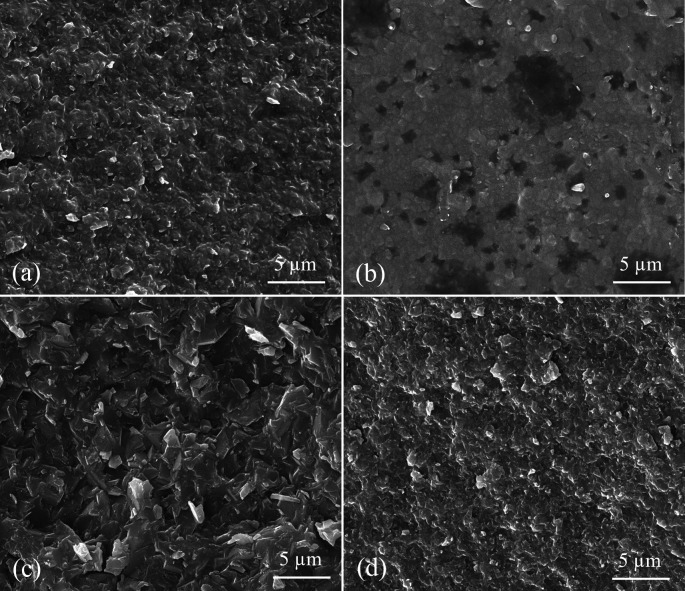
SEM representing surface morphology of tested CAD/CAM ceramics at x10000 magnification where (a) ALD; Advanced lithium disilicate (b) ZLS; Zirconia lithium silicate, (c) LD; Lithium disilicate, (d) LE; Leucite reinforced.

SEM images at ×10000 magnification showed the crystalline structure of ALD, ZLS, LD, and LE specimens ( Figure 3). ZLS showed a homogenous crystalline matrix ( Figure 3a). At the same time, LD had needle-shaped fine-grained crystals within a glassy matrix ( Figure 3b). LE and ALD images showed numerous pores with leucite crystals and lithium aluminum silicate crystals incorporated in a glassy matrix, respectively ( Figure 3c and 3d).

## Discussion

This study assessed the flexural strength of four CAD/CAM ceramic materials of varying thicknesses and compositions. The results showed that 1.5 mm thickness in the ZLS group was the highest flexural strength. Similar findings were noted in ZLS of 1.5 mm thicknesses in terms of elasticity. The highest strength was noticed among the ZLS specimens, LD, ALD, and LE, respectively, which subsequently required higher force to fracture. Specimens with a thickness of 1.5 mm exhibited significantly greater strength compared to the 0.5 mm samples, which presented the least strength. Thus, the null hypothesis is rejected.

Different thicknesses of ceramic material might yield varying flexural strength values on the ceramic materials. Ceramic materials of less thickness, such as 0.5 and 1mm, are suitable for minimally invasive procedures. ^31^ Schweiger J et al. ^32^ investigated three different types of CAD/CAM materials: LE, LD, and 3Y-TCP zirconia at five different thicknesses ranging from 0.4-1.6 mm. The lowest load required to fracture was recorded at the 0.4 mm thickness, while the highest load was required for the 1.6 mm thickness of zirconia, followed by LD and LE ceramics. This is consistent with the current study’s findings, where the 0.5 mm thickness of LE ceramics required the least force to fracture, while the 1.5 mm thickness of ZLS and LD required the highest force to fracture, respectively. The results of the flexural strength test of this study were compared to the readings of the biaxial flexural strength test described by Schweiger J et al. ^32^ due to the lack of similar studies assessing the relation between varying ceramic thickness and the flexural strengths characteristic.

In assessing the relation between the ceramic composition of tested ceramics and flexural strength feature, ZLS of all thicknesses exhibited the highest flexural strengths compared to LD and ALD, respectively. Meanwhile, LE ceramics exhibited the least flexural strengths among all tested materials at different thicknesses. This comes in agreement with Attar et al. ^33^ findings, which reported that zirconia-reinforced lithium silicate ceramics (Vita suprinity) of 2 mm thickness exhibited higher flexural strengths than LD and LE, respectively. Similar findings were stated by Elsaka et al. ^34^ after assessing the flexural strengths and elastic moduli of Vita suprinity and LD of 1.2 mm thickness. The higher strengths and elastic modulus of zirconia-reinforced ceramics are related to the presence of ZrO _2_ particles in the glassy matrix as in SEM images, resulting in a higher resistance to crack propagation. ^33, 34^ In contrast, Corade et al. ^35^ showed increased flexural strength of LD specimens of 1.5 mm compared to both ZLS and other zirconia reinforced lithium disilicate ceramics of different manufacturers (Vita suprinity and Rosetta). This might be referred to the different methods used in both studies.

The current results showed that LD reported higher flexural strength than ALD and LE ceramics. In agreement with these findings, another study reported that crystalized LD of 1 mm thickness exhibited higher flexural strength compared to ALD after exposure to different firing and glazing protocols. ^36^ Similarly, another study displayed that the highest flexural strength was reported among LD specimens of 3 mm thickness contrasted to ALD. ^16^ Furthermore, Sonmez et al. ^37^ found that LD specimens of 1.2 mm thickness showed higher flexural strength than those of LE ceramics. These superior properties of LD might be due to the difference in composition, where LD includes a tiny amount of glass phase and lithium disilicate crystals, as shown in SEM.

Strengths of this study included assessing the flexural strengths of the most used CAD/CAM ceramics, varying in thicknesses relevant to fabricating different esthetic restorations such as dental veneers, veneered restorations, and all-ceramic prostheses. Moreover, a thermocycling procedure was applied to simulate an aging process equivalent to 6 months intraorally. Additionally, the study evaluated the flexural strengths and topography of Cerec tessera (ALD) ceramics, a type of ceramic that is relatively new in the CAD/CAM realm and has not been extensively studied in the literature, especially in variable thicknesses.

Despite the strengths, the study has several limitations; one major limitation is that it is an in vitro study, which may not fully represent the complex oral environment. Therefore, further clinical studies are required to assess different restoration designs and a wider range of dental materials to better simulate oral conditions. Moreover, the study only assessed low translucency ceramics, and future studies should consider evaluating different levels of translucency to understand their impact on flexural strengths more comprehensively.

## Conclusions

The increase in ceramic thickness significantly impacts the flexural strength. A thickness of 1.5 mm was found to be optimum in restoring teeth in the posterior region or subjected to heavy occlusal load. Additionally, the composition of CAD/CAM ceramics has a crucial role in the flexural strength property. Dental ceramics, including zirconia fillers, are more resistant to deformation under masticatory loads than other glass ceramics. This was noted in ZLS ceramics. However, ALD requires further investigations to validate the current findings.

## Data Availability

All the data analyzed during the study are included in the article. FigShare: Flexural strength of ceramics data set. Ellakany, Passent (2024) https://doi.org/10.6084/m9.figshare.27095080.v1 This project contains the following underlying data:
• Flexural strength ceramics data.xlsx. figshare. Dataset. https://doi.org/10.6084/m9.figshare.27095080.v1 Data are available under the terms of the Creative Commons Attribution 4.0 International license (CC-BY 4.0).

## Software availability statement

IBM statistics software can be replaced by Open source Jamovi software free version.
