# Supplementary material for: Effect of thermocycling on flexural strength of dental CAD/CAM ceramics of variable thicknesses and structures: an in vitro study
Source: F1000Res. 2025 Aug 14;13:1310. Originally published 2024 Oct 31. [Version 3] doi: 10.12688/f1000research.157128.3 (PMC12322662; doi:10.12688/f1000research.157128.3)
Supplement: Peer review history

# Reviewer report: Reviewer response for version 3

**DOI:** 10.5256/f1000research.185985.r405529

**Published:** 2025-09-05

**Authors:** Gilan Altonbary

**Affiliations:** 1 Department of Removable Prosthodontics, Faculty of Dentistry, Mansoura University, Mansoura, Egypt

I think that the authors have adequately addressed the comments made by the reviewers in the revised version of the manuscript. Therefore, I have no further comments.

Is the work clearly and accurately presented and does it cite the current literature?

Yes

If applicable, is the statistical analysis and its interpretation appropriate?

I cannot comment. A qualified statistician is required.

Are all the source data underlying the results available to ensure full reproducibility?

Yes

Is the study design appropriate and is the work technically sound?

Yes

Are the conclusions drawn adequately supported by the results?

Yes

Are sufficient details of methods and analysis provided to allow replication by others?

Yes

Reviewer Expertise:

CAD/CAM , implant prosthodontics

I confirm that I have read this submission and believe that I have an appropriate level of expertise to confirm that it is of an acceptable scientific standard.

# Reviewer report: Reviewer response for version 3

**DOI:** 10.5256/f1000research.185985.r405530

**Published:** 2025-09-04

**Authors:** Rania Moussa

**Affiliations:** 1 Department of Substitutive Dental Science, College of Dentistry, Taibah University, Madinah, Saudi Arabia

I would like to thank the authors for addressing the previous comments and making the necessary adjustments. The revisions have significantly improved the clarity and quality of the manuscript. I acknowledge the efforts made, and based on the current version, I recommend the paper for indexing.

Is the work clearly and accurately presented and does it cite the current literature?

Partly

If applicable, is the statistical analysis and its interpretation appropriate?

Yes

Are all the source data underlying the results available to ensure full reproducibility?

Yes

Is the study design appropriate and is the work technically sound?

Yes

Are the conclusions drawn adequately supported by the results?

Yes

Are sufficient details of methods and analysis provided to allow replication by others?

Yes

Reviewer Expertise:

prosthodontic materials and dental implant restorations.

I confirm that I have read this submission and believe that I have an appropriate level of expertise to confirm that it is of an acceptable scientific standard.

# Reviewer report: Reviewer response for version 3

**DOI:** 10.5256/f1000research.185985.r405531

**Published:** 2025-08-25

**Authors:** Amr Rizk

**Affiliations:** 1 Prosthetic Dentistry, King Salman International University, El-Tur, South Sinai, Egypt

I would like to thank the authors for enhancing the overall quality of the manuscript. Please put the following in consideration.

1. The formula of lithium disilicate, the numbers should be in subscripts.
2. It was mentioned “Post-polishing, the pre-crystallized specimens were placed in a furnace of ceramic to crystallize”. That is incorrect as ALD comes in a crystallized form, although it still requires firing for the development of Virgilite.

Is the work clearly and accurately presented and does it cite the current literature?

Yes

If applicable, is the statistical analysis and its interpretation appropriate?

Yes

Are all the source data underlying the results available to ensure full reproducibility?

Yes

Is the study design appropriate and is the work technically sound?

Partly

Are the conclusions drawn adequately supported by the results?

Yes

Are sufficient details of methods and analysis provided to allow replication by others?

Partly

Reviewer Expertise:

Ceramics, color, translucency, marginal adaptation, internal fit, flexure strength, fracture resistance, dental materials and recent innovative restorations.

I confirm that I have read this submission and believe that I have an appropriate level of expertise to confirm that it is of an acceptable scientific standard.

# Reviewer report: Reviewer response for version 2

**DOI:** 10.5256/f1000research.183704.r395480

**Published:** 2025-08-04

**Authors:** Rania Moussa

**Affiliations:** 1 Department of Substitutive Dental Science, College of Dentistry, Taibah University, Madinah, Saudi Arabia

Dear authors, Thank you for your efforts. The research addressed the impact of ceramic material composition in varying thicknesses on the flexural strength and elastic modulus.

 This study is well-designed and addresses a significant question in restorative dentistry. It provides valuable comparative data on the flexural strength of current CAD/CAM ceramics after simulated ageing.

 The introduction is clear and well written.

 The methodology is clear and provides sufficient details.

**Results** are clearly presented. Tables and figures effectively summarise flexural strength, force at break, and Young’s modulus across groups and thicknesses. Statistical significance is clearly marked. However,

 Some parts in the results need adjustment:

 1- 2nd paragraph in the results: "while LE had the lowest ( **mean (SD) = 82.80 (12.35**)."this value is different from the data in table 1, please check and correct

 2-paragraph 3 in the results: The 0.5mm thickness across all ceramic groups had the lowest f **lexural strength,** **elastic modulu**s, and **forces to break; **the arrangement of the mechanical test does not match the arrangement of the values for the materials reported: "highest values (mean (SD) = **309.08 (33.49), 416.56 (74.55) and 82.80 (12.35)**), please revise and correct either the textual part or the order of the values.

 3- SEM results: the legends in the text are not matching the legends on Figure 3, e.g., 3a is not ZLS and so on for all the materials." ZLS showed a homogenous crystalline matrix ( **Figure 3a**). At the same time, LD had needle-shaped fine-grained crystals within a glassy matrix ( **Figure 3b**). LE and ALD images showed numerous pores with leucite crystals and lithium-aluminium silicate crystals incorporated in a glassy matrix, respectively ( **Figure 3c and 3d).**

 The discussion is well written, as it explains the results and compares them to the current literature; however,

 1- The study does not attempt to correlate flexural strength values with clinical performance or failure rates, which would aid in translating findings to practice. Please discuss the clinical relevance and relate flexural strength thresholds to actual clinical failure rates or guidelines, enhancing the practical value of the findings.

 2- 5000 thermocycles, corresponding to six months of clinical aging, is relatively a short period of functional use of a restoration, and this point should be highlighted in the limitations of the study

Is the work clearly and accurately presented and does it cite the current literature?

Partly

If applicable, is the statistical analysis and its interpretation appropriate?

Yes

Are all the source data underlying the results available to ensure full reproducibility?

Yes

Is the study design appropriate and is the work technically sound?

Yes

Are the conclusions drawn adequately supported by the results?

Yes

Are sufficient details of methods and analysis provided to allow replication by others?

Yes

Reviewer Expertise:

prosthodontic materials and dental implant restorations.

I confirm that I have read this submission and believe that I have an appropriate level of expertise to confirm that it is of an acceptable scientific standard, however I have significant reservations, as outlined above.

# Reviewer report: Reviewer response for version 2

**DOI:** 10.5256/f1000research.183704.r393621

**Published:** 2025-07-28

**Authors:** Amr Rizk

**Affiliations:** 1 Prosthetic Dentistry, King Salman International University, El-Tur, South Sinai, Egypt

This manuscript entitled “Effect of thermocycling on flexural strength of dental CAD/CAM ceramics of variable thicknesses and structures: an in vitro study" aims to compare mechanical properties of glass ceramics of varying composition and thickness. The topic is relevant and falls within the scope of the journal.

 Overall, the manuscript is well-written and excuted. However, several points require clarification as detailed below.

 Title:

1. In the title it was mentioned thermocycling and again in the abstract it was mentioned that samples were subjected to thermomechanical cycling and again in the methodology in figure 1, it was mentioned thermocycling. Both are different please be concise and adjust accordingly.

Introduction:

1. It was mentioned that “Advanced lithium disilicate (ALD), a recent addition to the market”. Actually, it is not recent anymore being introduced over more than 3 years. Please adjust accordingly.
2. “(Li2Si2O5)” this is incorrect formula for lithium disilicate. Please revise as numbers are subscript.
3. Please tell the readers what is Virgilite formula.

Materials and methods:

1. In figure 1, add to each material n=30 and total 120 samples, add the number of thermomechanical/thermocycles and the magnification used for SEM.
2. It was mentioned that “Post-polishing, the specimens were placed in a furnace of ceramic to crystallize.”. This is not correct as some of the materials are fully crystallized although still require firing.
3. Were samples glazed and what is the reason for post firing polishing?
4. Again what are the parameters for the mechanical cycling? You only mentioned the thermal part.
5. “Flexuralstrength= 3FL/2bh2 is that formula correct”. Please add space between flexure and strength and write it using equation editor in MS word.
6. Do the same in elastic modulus formula.
7. Please add that measurements for L, b and h are in mm.

Results:

1. “Among the 1.5 mm thicknesses, ZLS had the highest Young’s modulus of elasticity (mean (SD) = 82.80 (12.35), while LE had the lowest (mean (SD) = 82.80 (12.35).” at the end of both presented numbers a “)” is missing to be consistent in all of the manuscript.
2. In figure 3, there are no apparent red arrows as mentioned by the authors.

Discussion:

1. It was mentioned “The results of the flexural strength test of this study were compared to the readings of the biaxial flexural strength test described by Schweiger J et al.32 due to the lack of similar studies assessing the relation between varying ceramic thickness and the flexural strengths characteristic.” Please review this study as it assessed the biaxial flexure strength of ALD with a thickness of 0.5 and 1-mm. DOI: 10.1007/s00784-024-05746-8
2. Again, what did the authors adopt thermomechanical or thermocycling?
3. If samples were not glazed, that might be considered a limitation, together with the lack of cementation.

Is the work clearly and accurately presented and does it cite the current literature?

Yes

If applicable, is the statistical analysis and its interpretation appropriate?

Yes

Are all the source data underlying the results available to ensure full reproducibility?

Yes

Is the study design appropriate and is the work technically sound?

Partly

Are the conclusions drawn adequately supported by the results?

Yes

Are sufficient details of methods and analysis provided to allow replication by others?

Partly

Reviewer Expertise:

Ceramics, color, translucency, marginal adaptation, internal fit, flexure strength, fracture resistance, dental materials and recent innovative restorations.

I confirm that I have read this submission and believe that I have an appropriate level of expertise to confirm that it is of an acceptable scientific standard, however I have significant reservations, as outlined above.

## References

[1] : Effect of multiple firings on optical and mechanical properties of Virgilite-containing lithium disilicate glass-ceramic of varying thickness. *Clinical Oral Investigations* .2024;28(7) : 10.1007/s00784-024-05746-8 10.1007/s00784-024-05746-8 PMC1117622938869624

# Reviewer report: Reviewer response for version 1

**DOI:** 10.5256/f1000research.172537.r352365

**Published:** 2025-03-27

**Authors:** Joissi Ferrari Zaniboni

**Affiliations:** 1 São Paulo State University, São Paulo, Brazil

Dear Editor,

 Thank you for inviting me to review the article. Count on me for the next ones.

 The topic is relevant, especially because of the use of a material that has not been explored much in the literature and also in clinical practice, such as Tessera. I think that what was missing in this study, to enrich it, is a comparison of the values ​​before and after thermocycling so that we can judge whether the aging process interferes with the material's performance or not.

 In addition, I suggest some changes:

**1)** Results in the Abstract: "The lowest flexural strength was observed in the 0.5 mm thickness group." Specify whether it was observed in all materials or in some specific materials.

**2)** In the Result section: "while had the lowest (mean (SD)= 82.80 (12.35)". Identify the LE group and correct the data.

**3)** Be careful to say that ZLS presented the best results, in relation to only the LE material, since there was no statistically significant difference in the others.

**4)** I don't see the need to present Table 2, since in Table 1 it is already possible to see that there is interaction between the factors and which groups have a significant difference or not. Therefore, I do not see the need for Figure 2.

**5)** In Figure 3, I suggest indicating with arrows where the pores are present.

**6)** Although, in the limitation of the study it was mentioned that they should evaluate the other translucency of the materials, I think that a limitation was also not evaluating the translucency in the different thicknesses of the current work. This could be additional data to add to the results of the work.

Is the work clearly and accurately presented and does it cite the current literature?

Partly

If applicable, is the statistical analysis and its interpretation appropriate?

Yes

Are all the source data underlying the results available to ensure full reproducibility?

Partly

Is the study design appropriate and is the work technically sound?

Partly

Are the conclusions drawn adequately supported by the results?

Yes

Are sufficient details of methods and analysis provided to allow replication by others?

Yes

Reviewer Expertise:

Ceramics, CAD-CAM, adhesion

I confirm that I have read this submission and believe that I have an appropriate level of expertise to state that I do not consider it to be of an acceptable scientific standard, for reasons outlined above.

# Reviewer report: Reviewer response for version 1

**DOI:** 10.5256/f1000research.172537.r339059

**Published:** 2024-12-10

**Authors:** Gilan Altonbary

**Affiliations:** 1 Department of Removable Prosthodontics, Faculty of Dentistry, Mansoura University, Mansoura, Egypt

the authors are to be complimented for conducting this study. Its design is very good and materials and methods appropriate ,

  in the introduction section the authors mentioned that  research reporting the mechanical properties of ALD is scarce however an invitro study by Freitas et al 2023 [Ref 1] investigated surface properties and fatigue mechanical behavior of  advanced lithium disilicate (ALD), and  compared it to a conventional lithium disilicate (LD) and to a translucent polycrystalline zirconia-based ceramic (4Y-PSZ) this study can also be included in the discussion section as it reported that (ALD) has lower roughness among the tested materials (lithium disilicate and yttrium-stabilized zirconia ceramics). Regarding flexural fatigue strength, ALD presented lower values and greater variability in mechanical performance (resulting in a lower structural reliability) when compared to the other materials 

 in the conclusion section the authors stated that the increase in ceramic thickness significantly impacts the flexural strength according to the study results, however this was not fully addressed in the discussion; as the increase thickness of material  from 0.5 to 1.5 mm lead to increase transformation toughness, Also, greater the thickness will decrease the bending force in ceramic material which explain increasing in flexural strength with higher thicknesses

 another limitation for this invitro study is the specimens shape and design( please mention in the methodology section), which do not mimic the geometry of crown-bridge restorations.

Is the work clearly and accurately presented and does it cite the current literature?

Yes

If applicable, is the statistical analysis and its interpretation appropriate?

I cannot comment. A qualified statistician is required.

Are all the source data underlying the results available to ensure full reproducibility?

Yes

Is the study design appropriate and is the work technically sound?

Yes

Are the conclusions drawn adequately supported by the results?

Yes

Are sufficient details of methods and analysis provided to allow replication by others?

Yes

Reviewer Expertise:

prosthodontics, CAD/CAM

I confirm that I have read this submission and believe that I have an appropriate level of expertise to confirm that it is of an acceptable scientific standard.

## References

[1] : Surface properties and flexural fatigue strength of an advanced lithium disilicate. *J Mech Behav Biomed Mater* .2023;147: 10.1016/j.jmbbm.2023.106154 106154 10.1016/j.jmbbm.2023.106154 37804677
